# COVID-19 AND DENTAL DISTANCE-BASED EDUCATION: students’ perceptions in an Italian University

**DOI:** 10.1186/s12909-021-02840-3

**Published:** 2021-08-02

**Authors:** Paolo Di Giacomo, Carlo Di Paolo

**Affiliations:** Department of Oral and Maxillo-facial Sciences, Sapienza University of Rome, Via Caserta 6, 00161 Rome, RM Italy

**Keywords:** Distance-based education, Dentistry, COVID-19 pandemic

## Abstract

**Background:**

The aim of the study was to analyze the perception of dental faculties students regarding the complete transition to distance-based education (DE) and the adaptation of this educational strategy, due to Covid-19 pandemic. A questionnaire to be completed anonymously was submitted online to students attending the faculties of Dentistry and Oral Hygiene at Sapienza, University of Rome, after the end of distance lessons. The collected data were processed statistically, providing descriptive data and analysis of correlation of the most significant parameters, using Chi-squared test, Cramér V and Pearson φ^2^, Goodman and Kruskal’s γ and λ and Kendall’s τb. The level of statistical significance was *p* < 0.05.

**Results:**

A total of 314 students participated in the survey. The overall level of satisfaction on a ten- point scale was 5.39 ± 2.59 for Oral Hygiene students and 6.15 ± 2.98 for Dentistry students. The most common complaints were the lack of a structured online curriculum, less interaction with professors and a lower level of attention. On the basis of the responses, scored using Likert-type Scale, oral Hygiene students reported statistically higher level of physical fatigue(*p* = 0.0189), a lower level of attention (*p* = 0.0136) and of the quality and quantity of acquired knowledge during distance education (*p* = 0.0392), compared to Dentistry students. Level of perceived stress and quality and quantity of acquired knowledge (γ = 0.81 and τb =0.56) and quality and quantity of acquired knowledge and fear of a decrease in knowledge (γ = 0.76 and τb =0.54) are associated variables.

**Conclusion:**

Students’ feedback is essential to solve the key issues emerged from the questionnaire. New educational models should be define in order to ensure that distance education could be effective, meeting the learning needs of the students, and could not be a merely “online shift” of traditional methods, used as an alternative of live education.

**Supplementary Information:**

The online version contains supplementary material available at 10.1186/s12909-021-02840-3.

## Background

The COVID-19 pandemic is the threat and global emergency affecting the World public health in 2020, with an extensive and devastating impact [1]. It has led to a sudden and radical change of life of the worldwide population, forced to physical distancing and home isolation by National Governments, for the sake of limiting the widespread outbreak. Due to the restrictions recommended by the Centers for Disease Control and Prevention and other organizations, the employment and educational landscapes are rapidly changed.

The complete transition toward a distance-based education involved all educational levels, testing the limited Italian experience in this field. Many critical issues emerged in relation to the students’ age, the type of curriculum and the availability and management of technological sources.

As for medical education, the impossibility to conduct in-person education led many States to a call for action in order to ensure continuity of teaching in various fields of medicine [2–6]. However, in order not to expose medical students to the risk of contagion, clerkships were suspended. This event raised many concerns about the professional training of future doctors.

As for dental education, since 5 March 2020, Italian Universities have quickly adapted, cancelling in-person medical classes and clinical rotations, which were replaced by digital lessons and distance education. In Italy, Dentistry is a six-year course and Oral Hygiene is a three-year course. In the former, the pre-clinical curriculum involves mainly the first 5 years, characterized by traditional in-person classes. Clerkships and theoretical lessons are planned during the last academic year. In the Oral Hygiene course, theoretical lessons were held during the first 2 years and practical activities mainly during the last one. During pandemic, even if the study schedule of the students, who were not attending the last year of their course, did not change from a “theoretical perspective”, the shift to distance education represented a big change. The education of the students who were close to graduation underwent an even more radical change, due to the suspension of clerkships.

The biggest concern of academics was the loss of active interaction as a potential damage to education and study, both from a theoretical and practical point of view. In fact, the irreplaceable value of attending class in-person, lauding the real-time feedback and interaction with professors and peers, useful for learning and developed in class [1] have been emphasized for all levels of education. Furthermore, the practical clinical activities as well, which characterize the curricula of medical faculties, represent a crucial moment of medical education. However, while no alternatives to practical training were possible, distance education, especially for theoretical topics, has been proved to be efficient in different educational and governmental studies, supported by more and more performing technologies [7–13]. However, the availability of efficient institutional strategies represents a major challenge for integrating distance learning in medical education [14, 15].

The key aspects to be considered are the abruptness and radicalism of the shift towards a different and unfamiliar type of education for teachers and students, within an unprecedented context, such as COVID-19 pandemic. This fact has been a challenge in terms of teaching strategies, availability of adequate technological sources and students’ attitude to distance learning. Furthermore, such pandemic context represented a stressful event with a negative impact on people’s mental health. Covid-19 related stressors and associated mental and emotional disorders (anxiety, depression, insomnia) might have led to an even more difficult adaptation to distance education and made this educational transition truly unique, both for teachers and students [ 16, 17].

During lockdown, distance-based education of dental faculties at Sapienza University has been characterized by online lessons through a digital platform, according to the lesson schedule planned at the beginning of the semester. Before pandemic, online lectures and webinar were not being held and the institutions were unfamiliar with digital platform to conduct them. Instead, already before the pandemic, Sapienza University provides e-learning tools, such as the free and open-source learning management system “Moodle (the acronym of Modular Object-Oriented Dynamic Learning Environment), where the students could have information and additional study materials in relation to their course.

Distance-education was born before Covid-19 pandemic with a well-defined structure, so the application of traditional teaching methods in distance learning might be an unproper choice [18].

In this context, the authors aimed at analyzing the feedback of students attending dental faculties of Sapienza University of Rome, on their distance-based education during lockdown, also in order to improve the quality of any future online experience in that University. In particular, the study aimed at analyzing technology availability and usability, use of interactive methods in teaching and learning the pre-clinical curriculum, level of knowledge, students’ attitude and behavior (concentration, fatigue, etc).

## Materials and methods

### Study design

This study was performed at Sapienza University of Rome in June 2020, at the end of online lesson schedule. The students’ perception collected through the questionnaire is related to the period between March and June 2020, when the University used video-live lessons on digital platforms, in order to replace face-to-face teaching.

An online questionnaire was created as data collection tool, designed by the authors specifically for this research, as reported in the Additional file 1 and was submitted to the attention of a representative sample of students attending the faculties of Dentistry and Oral Hygiene at Sapienza, University of Rome.

The elaboration of the questionnaire took its cue from the scientific literature [19–26]. The original version of the questionnaire was piloted among a group of students to ensure suitability, validity, practicability and interpretation of answers. On the basis of the comments and suggestions obtained, the questionnaire was revised.

The final version consisted of 24 open and close-ended questions. Four questions helped to define the student profile (age, sex, type and year of the course). Three questions were about means such as environment and suitability of the platform. Seven questions were on the quality of lessons and methods used. Three were about the impact on mental and physical sphere (stress perceived, physical fatigue, degree of attention). Two compared the in-class lessons and the distance-based ones. Two were about students concerns and level of satisfaction. Three were about suggestions and positive and negative aspects of distance learning.

The questionnaire was uploaded online to the free survey platform GOOGLE SURVEY (surveys.google.com, Google LLC) and the link was sent to the students’ institutional emails. Students were informed about the study and asked to complete the questionnaire online. An informative text was inserted at the top of the questionnaire and informed consent was obtained in the form of students stating their agreement to participate in the study. The data collected were anonymous, and tracing the identity of the subjects was not possible.

All methods were carried out in accordance with relevant guidelines and regulations. The study was approved by the Institutional Ethics Committee of Department of Oral and Maxillo-Facial Sciences of Sapienza University of Rome (Protocol no. 0001278 47/2020).

### Study population and sampling strategies

Starting from the total number of the students attending Dentistry and Oral Hygiene at Sapienza, the authors applied the Cochran’s formula to define the adequate sample size. Sample size was calculated based on a response rate of 50%, a confidence interval of 95% and a margin of error of 5%, with a total student population of approximately 630 subjects. The largest required sample size was 240 students.

### Statistical analysis

In this study, descriptive statistical analysis (percentage, average, median, mode, standard deviation, minimum, and maximum value) was carried out. Several measures of association were performed including, Chi-squared test, Fisher’s exact, Pearson φ^2^, Cramer’s V, Goodman and Kruskal’s γ and λ and Kendall’s τb [27–30]. The level of statistical significance was set at 0.05. The software used is STATA 15.1 (StataCorp LLC, TX, USA).

Chi-squared is a test for the independence of the rows and columns. The null hypothesis (H0) is that there is no relationship. To reject this we need a *P* < 0.05 (at 95% confidence). Pearson’s φ^2^ and Cramér’s V are measures of association between two nominal variables. Cramér V goes from 0 to 1, where 1 indicates strong association. Such tests were used to analyze the differences and statistical associations between the two medical faculties and between genders as for the answers given by the students to the questions no. 13-14-15 and 17-18-19-20.

γ and λ by Goodman & Kruskal’s and Kendall’s τb are measures of association between two variables. Both go from − 1 to 1. Negative shows inverse relationship, closer to 1 a strong relationship. γ is recommended when there are lots of ties in the data and τb is recommended for square tables. λ is a measure of proportional reduction in error in cross tabulation analysis.

Such tests were used to analyze the degree of association between:

1. Ability of the teacher in stimulating interest and student’s level of attention;
2. Possibility to interact with the teacher and student’s level of attention;
3. Level of perceived stress and quality and quantity of the acquired knowledge;
4. Quality and quantity of the acquired knowledge and fear of a reduction of the own level of knowledge.

Student t-test was performed to compare the average values of the groups males/females and Dentistry/Oral Hygiene students as for the level of overall satisfaction.

## Results

The link of the survey was sent to 630 students. The sample who agreed to participate was composed by 314 students, 240 females (75.9%) and 37 males (24.1%), with a mean age of 24.5 years (range 19–45 years).

One hundred eighty-four students (58,59%) attended Oral Hygiene (86 students – first year, 76 – second year, 22 – third year), 130 students (41,41%) Dentistry (18 –first year, 6 s year, 10 – third year, 6 –forth year, 60 – fifth year, 30 – sixth year).

**Fig. 1:**
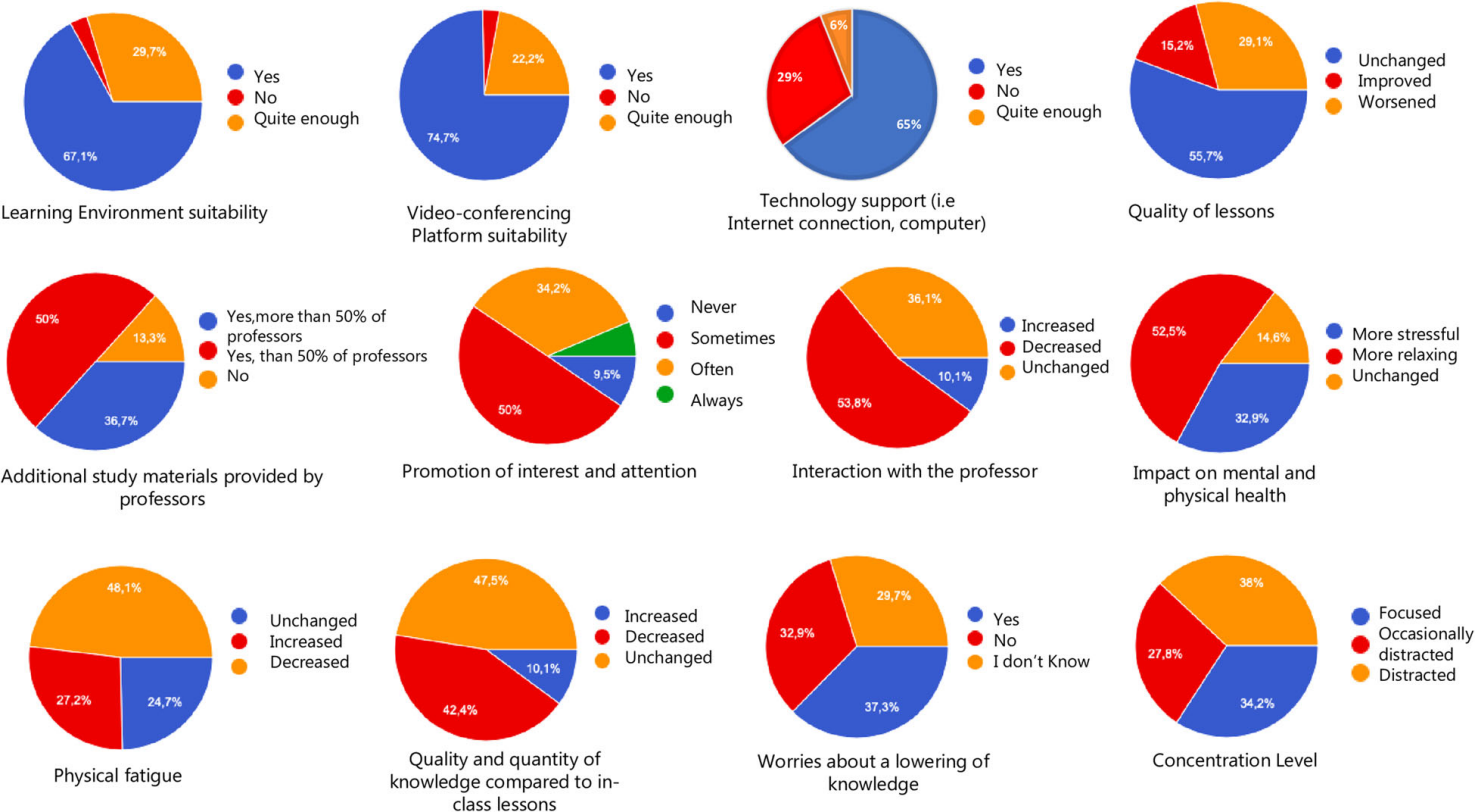
Pie-charts of the descriptive analysis

The analysis of the answers were reported in the following graphs (Fig. 1**and** Table 1, 2, 3, 4, 5).

**Table 1:** Mean scores ± standard deviation (SD) for the questions number 9-10-11-14

Question No.	Mean Score ± SD
No 9. Frequency of traditional lessons	3.60 ± 1.26 (on a five point scale)
No 10. Frequency of flipped classroom	1.05 ± 1.35 (on a five point scale)
No 11. Frequency of problem solving	2.22 ± 1.35 (on a five point scale)
No 14. Hours/week of distance lessons	11.78 h/ week

**Table 2:** Report of the answers for questions no. 21-22-23-24

**Question No. 21** “**What is your overall level of satisfaction level in distance learning?”.**	
Total sample 5.48 ± 2.75	
Oral Hygiene 5.39 ± 2.59 Dentistry 6.15 ± 2.98 *p* value 0.09 not significative	
Females 5.54 ± 2.618 Males 5.29 ± 3.17 p value 0.63 not significative	
**Question No 22. “What would you change to improve your online lessons?”**	
Most frequent answers:	
- More problem solving	
- Receiving didactical materials before lessons	
- More interaction	
- Better internet connection system	
**Question No. 23. “What is the best aspect of online lessons?”**	
Most common answers:	
- More comfort	
- Reduction of travel- related stress	
**Question No.24 “What is the worst aspect?”**	
Most common answers:	
- Internet connection problems	
- More distraction	
- Spending a lot of time in front of the computer

**Table 3:** Measures of association-gender. Gender is the variable assessed, in relation to the answers about the quality of lessons, the promotion of attention, the perception of stress, the physical fatigue, the concentration, the interaction, the level of acquired knowledge and concerns

Gender- question no.	Chi-squared test	Cramer V	Pearson’s ϕ^2^
No 8. Quality of lessons	2.39 (*p* value 0.30)	0.19	0.036
No 13. Promotion of attention	1.25 (*p* value 0.74)	0.10	0.01
No 15. Perception of stress	0.39 (*p* value 0.53)	0.031	0.001
No 16. Physical fatigue	0.31 (*p* value 0.85)	0.024	0.0006
No 17. Concentration	1.45 (*p* value 0.48)	0.116	0.013
No 18. Interaction	2.43 (*p* value 0.29)	0.195	0.038
No 19. Level of Knowledge	4.22 (*p* value 0.63)	0.339	0.115
No 20. Concern about knowledge	0.92 (*p* value 0.22)	0.074	0.0054

**Table 4:** Measures of association- type of faculty. The variable assessed is type of course (Dentistry/Oral Hygiene), in relation to the answers about the quality of lessons, the promotion of attention, the perception of stress, the physical fatigue, the concentration, the interaction, the level of acquired knowledge and concerns

Faculty- question no.	Chi-squared test	Cramer V	Pearson’s ϕ^2^
No 8. Quality of lessons	5.65 (*p*value 0.06)	0.45	0.20
No 13. Promotion of attention	0.47 (*p*value 0.49)	0.037	0.0014
No 15. Perception of stress	4.74 (*p*value 0.093)	0.38	0.145
No 16. Physical fatigue	7.94 (*p*value **0.0189)**	**0.637**	**0.406**
No 17. Concentration	8.60 (*p*value **0.0136**)	**0.69**	**0.477**
No 18. Interaction	2.23 (*p*value 0.32)	0.18	0.03
No 19. Level of Knowledge	6.48 (*p*value **0.0392**)	**0.52**	**0.27**
No 20. Concern about knowledge	2.99(*p*value 0.22)	0.24	0.057

Bold type = statistically significant.

**Table 5:** Measures of association between questions. Bold type = statistically significant

Associated variables	Goodman and Kruskal’s ***γ***	Goodman and Kruskal’s ***λ***	Kendall’s ***τ***b
1. Ability of the teacher in stimulating interest and student’s level of attention;	0.46–0.50	0.20	0.30
2. Possibility to interact with the teacher and student’s level of attention;	0.46	0.20	0.30
3. Level of perceived stress and quality and quantity of the acquired knowledge;	**0.81**	**0.46**	**0.5587**
4. Quality and quantity of the acquired knowledge and fear of knowledge reduction.	**0.76**	**0.36**	**0.5419**

There are not statistically significant differences between males and females as for questions no.8,13, 15-20. (*p*-value < 0.05), as shown in the Table 3.

There is a statistically significant difference as for question no. 16 “At the end of the day, compared to in-class lessons, physical fatigue is ….” between students attending Hygiene classes and Dentistry ones (χ^2^ = 7,92; 2 df). Physical fatigue is more perceived by Hygiene students.

There is a statistically significant difference as for question no. 17 “During online lessons …” , where Oral Hygiene students referred to be more distracted (χ^2^ = 8.60, 2 df).

There is a statistically significant difference as for question no. 19 “The quality and quantity of acquired knowledge ….” between students attending Hygiene classes and Dentistry ones (χ^2^ = 6,48; 2 df). Hygiene students stated a worsening of the acquired knowledge as for quality and quantity, compared to dentistry students, which considered it unchanged, as shown in the Table 4**.**

As for the association between variables, a) the level of perceived stress and b) the quality and quantity of acquired knowledge showed a significant association as well as a) the quality and quantity of acquired knowledge and b) the fear of a reduction of the own level of knowledge **(**Table 5**)**.

## Discussion

The study aimed at evaluating students’ feedback on their experience of distance-based education, which has proved to be essential in ensuring the continuity of teaching during the Covid-19 pandemic.

The forced closure of Italian Schools and Universities hastened the transition to distance education via digital platforms. For over a decade, medical faculties had enhanced the use of technology, making lessons more interactive [6, 31]. But, actually, as for this University, distance education was considered as an almost totally new approach for teachers and students of dental faculties. In this pandemic context, the University organizations made a great effort to be able to adapt to it. Each University has organized itself autonomously, and even within the same structure, each professor has applied his/her own education method. On one hand, this could have ensured a variety of sources and educational approaches. On the other one, without an outlined didactic planning, this could have led to a failure to develop the proper “online teaching skills”, mining the quality standard of the education.

In this study, the authors analyzed students’ opinions in relation to the following categories: a) learning environment and technologies; b) online courses/programs planning and development of an interactive and collaborative student-centered education; c) students’ suggestions and concerns. Such categories were analyzed in order to identify strengths and critical issues.

As for point a) is concerned, students reported that internet connection problems (29-35%) and the presence of not fully performing platforms for video and audio conferencing (3-25%) made distance education difficult to manage. The learning environment, intended as the physical location where the students could learn was considered adequate. In fact, among the positive aspects of distance learning during lockdown, the students reported that learning in the home was more comfortable. The term learning environment may also include other virtual spaces such as the learning management systems. Even if there was not the presence of specific questions on this type of “digital learning environment”, positive feedback emerged from the open-ended question about the advantages of distance-based learning, such as for Moodle. Moodle is a free and open-source learning management system, which have introduced new possibilities in education which supplement face-to-face classrooms with distance education, in use for a few years at Sapienza University. Web 2.0 brought a new generation of Web development that allowed more social networking, folksonomy (a system for classifying, tagging, and categorizing content), interoperability (the ability of an electronic device to interact with another electronic device), collaboration, and communication [32]. However, Institutions should work on the implementation of educational technologies, using financial resources to develop online programs, and on the overcoming of the “gap in technology fields” of the most reluctant professors in the acquisition of new digital skills [33].

As for point b) is concerned, as reported by the students, the entire pre-clinical curriculum was put in an online format with a prevalence of more “traditional” lessons which are typical of face-to-face instruction, without considering the specific requests of a more interactive online learning. For example, flipped classroom was among the strategies proposed but with a very low frequency. Furthermore, flipped classroom was among the methods which the students preferred and expressly requested to improve their learning. As for clinical case-based learning, the frequency was higher than flipped classroom but lower than traditional lessons. On one hand, even if with the above mentioned limits of the platform, the University enhanced the use of live-video conferencing, as suggested by Virginia Gewin [34, 35]. In fact, web-lessons and videoconferences were the most popular tools used and they were performed on dedicated platforms [4, 36]. On the other one, as shown also by Gewin [34, 35], it is recommended to integrate online distance lessons with a series of didactic activities, also managed on dedicated platforms which offer greater chances of interaction and attention, respecting the privacy and the management of the student data. In 2004, Drs. Cook and Dupras published an article explaining the most effective way to create an online learning platform to be used in medicine. They highlight the importance of a user-friendly website design that is well maintained, as well as the integration of self-assessment features to learners’ engagement [34, 37]. However, a positive finding concerns the access to supplementary materials which was provided by most of the professors. The authors found that, compared to the previous face-to-face schedule, the number of lesson hours per week was much lower (11.78 h/ week). In fact, according to 2019 curriculum plan, the number of hours per week was on average 20 h per week for the students attending Dentistry and 18 h per week for Oral Hygiene students. This fact was likely due to difficulties in online reorganization caused by the unexpected and sudden closure of academic institutions.

This analysis shows that the primary purpose of academics in distance education should be to transform the apparent distance and the impossibility of social interaction given by the computer, intended as a barrier, into an open space with an unlimited access to information, knowledge and social connection, where the students can build their knowledge with personalized times and modes. More recently, among the options for improving digital education, it is reasonable to highlight the use of the approach of “flipping” the classroom or of blended lessons, already used in traditional teaching [6]. Such approach supported by technologies, is characterized by students’ access to information “anywhere, anytime, as often they desire” [7, 32, 38], for example through pre-class video lectures, after discussed with the professor. It could improve the educational environment, enhancing the participation of the students and the quality of the acquired knowledge [39]. Small-group case-based learning and team-based learning, used in the flipped classroom, are widely recommended because they increase the student motivation and reinforce their level of interest. E-learning material also has proved to enable parallel self-learning [4]. The combined use of such methods could implement both asynchronous and synchronous educational activity. Another option is peer-teaching, recognized as a powerful tool in pedagogy [4]. However, only implementing technology, students can develop collaborative skills and improve adaptability. The rationale of these different pedagogical approaches is the concept of student-based learning. According to this model, knowledge is not a merely delivering of information from the professor to the student, but rather is a personal construction of the learner.

The context of the pandemic and related stressors, may have made this process even more complex and difficult both for the students and teachers. Hence the importance of a greater “systematization” of online teaching methods, which still appeared not structured (question n 22 and 24), and the necessity to set out more clearly the means to achieve educational targets. Even if the quality of lessons seemed not to be significantly changed for these students, the change in the communication channel without an adequate adaptation of teaching strategies could have led to a loss of attention and interaction. Other factors such as anxiety, fear and depression generated by the context of the pandemic may have played a role in this phenomenon [16, 17]. In particular, Oral hygiene students seemed to be more distracted and fatigued than Dentistry ones. No differences between genders as for perception (quality of lesson, promotion of attention, concerns, etc) and behaviors (such as concentration and fatigue) related to distance education.

Therefore, the overall level of satisfaction was on average not sufficient for Oral Hygiene students and just sufficient for Dentistry ones. Furthermore, 67% of students reported that they were worried or uncertain about the perspective of a lowering of the acquired knowledge by continuing distance-based education, whereas 42% stated a worsening of quality and quantity of the acquired knowledge (with a statistically significant difference between Oral Hygiene and Dentistry students) during lockdown online schedule, compared to the traditional one. This fact takes on even more value considering the students close to graduation who could not carry out internships, fundamental to consolidate and deepen the knowledge learned in the pre-clinical curriculum. An institutional support is essential for the success of distance education, and an institutional strategy should be designed to facilitate the implementation of key skills by faculty [40].

These variables can also be associated with each other, as shown by the statistics: students with an higher level of perceived stress reported a decreasing in the quality and quantity of the acquired knowledge and students who reported a decreasing in the quality and quantity of acquired knowledge were worried about the reduction of their own level of knowledge, by continuing Distance Education in the future.

As for the point c), the reported advantages of distance-based learning compared to in-class lessons were: a greater availability of time and greater comfort (52.5% of students were less stressed and 48.1% had less physical fatigue), considering that many students spend a lot of time in city travels; the possibility not to curb classes when unable to attend them in person. As for the disadvantages, in addition to the ones already highlighted, it is worth mentioning the greater possibility of distraction (38% reported to be distracted and 27.8% reported to be occasionally distracted) reported especially by Oral Hygiene students. Another critical aspect, which was the major complaint of the upper-level students close to conclude their studies, was the suspension of internships. Unfortunately, direct clinical experience and learning cannot be replaced by distance-education. However, especially in these “extreme conditions”, technological innovation could fill this gap as far as possible for example with virtual patients and virtual-reality simulators [41]. However, such alternatives have many limitations due to the practical aspects which characterized clerkships.

The results obtained are probably influenced by multiple components and the interpretation of data is more complex, because of the unique social contingency in which the distance education was experienced. The rapid evolution of the pandemic brought both students and faculty face to face with a less familiar educational method, since La Sapienza used the e-learning platform (i.e. Moodle) as the only distance learning tool. However, the possibility of conducting classes or entire courses online has never been contemplated. In the past, distance learning played a marginal role within an absolutely traditional educational context, instead, during pandemic, distance education was the only means available. Faced with this change, professors were not ready and they tried to adapt their approaches as much as possible, considering the resources and time available. However, the methods proposed were more appropriate for traditional teaching than for distance learning where interactive aspects are fundamental. On the other hand, the students, removed abruptly from their usual learning spaces, found it difficult to manage certain aspects related to distance learning, such as the absence of interaction with the teacher and their peers, with repercussions for example on concentration levels. Furthermore, factors such as the stressors related to the pandemic context, home isolation, the great amount of time spent in front of the computer have influenced the students’ perception. These factors probably affected also the students’ perception about their level of knowledge and concerns about the future.

As regards future perspectives, also in view of a possible transition to a hybrid education [42] due to the fluctuating trend of the pandemic, the critical issues identified by the students should be solved. In order to ensure that this type of approach is effective, meets the learning needs of the students and is not a merely “online shift” of traditional methods, it requires alignment with contemporary learning theory, implementing technology and developing teaching skills in order to support hybrid teaching and learning [42].

## Conclusion

Students’ feedback is essential to solve the key issues emerged from the questionnaire. The complete transition to the remote education in the wake of the Covid-19 emergency represented a challenge for teachers and students, due to factors related to the unique context and because of the adaptation of teachers and students to a “new” educational approach. The criticalities that emerged from this study, combined with the positive aspects, may allow the development of new, more adequate indications that can improve the online experience of both categories. This challenge will lead the academics to redefine new educational models which do not see the online teaching as “the alternative” to live teaching, i.e. during an emergency state, but also as an essential and integral part of a new educational curriculum, exploiting distance-based learning/teaching advantages.

## Supplementary Information

**Additional file 1.** Covid-19 and distance learning questionnaire.

## Data Availability

The authors confirm that the data supporting the findings of the study are available within the article and the datasets used and/or analysed during the current study are available from the corresponding author on reasonable request.

## References

[1] Ferrel MN, Ryan JJ (2020). The impact of COVID-19 on medical education. Cureus..

[2] Newman NA, Lattouf OM (2020). Coalition for medical education-a call to action: a proposition to adapt clinical medical education to meet the needs of students and other healthcare learners during COVID-19. J Card Surg.

[3] Aker S, Mıdık Ö (2020). The views of medical faculty students in Turkey concerning the COVID-19 pandemic. J Community Health.

[4] Kanneganti A, Lim KMX, Chan GMF, Choo SN, Choolani M, Ismail-Pratt I, Logan SJS (2020). Pedagogy in a pandemic - COVID-19 and virtual continuing medical education (vCME) in obstetrics and gynecology. Acta Obstet Gynecol Scand.

[5] Ahmed H, Allaf M, Elghazaly H (2020). COVID-19 and medical education. Lancet Infect Dis.

[6] Rose S (2020). Medical student education in the time of COVID-19. JAMA..

[7] Albarrak A. Education in a technological world: Communicating current and emerging research and technological efforts [Internet]. 1st ed. FormatexResearch Center; 2011.

[8] Letterie GS (2003). Medical education as a science: the quality of evidence for computer-assisted instruction. Am J Obstet Gynecol.

[9] Rotimi O, Orah N, Shaaban A, Daramola AO, Abdulkareem FB (2017). Remote teaching of histopathology using scanned slides via skype between the United Kingdomand Nigeria. Arch Pathol Laboratory Med.

[10] Bernard RM, Abrami PC, Lou Y, Borokhovski E, Wade A, Wozney L (2004). Howdoes distance education compare with classroom instruction? A meta-analysis of the empirical literature. Rev Educ Res.

[11] Liu Q, Peng W, Zhang F, Hu R, Li Y, Yan W (2016). The effectiveness of blended learning in health professions: systematic review and meta-analysis. J Med Internet Res.

[12] George PP, Papachristou N, Belisario JM (2014). Online eLearning for undergraduates in health professions: a systematic review of the impact on knowledge, skills, attitudes and satisfaction. J Glob Health.

[13] Cook DA, Levinson AJ, Garside S, Dupras DM, Erwin PJ, Montori VM (2008). Internet-based learning in the health professions: a meta-analysis. JAMA..

[14] Panahi P, Borna F. "Distance learning: challenges, new solution," 2014 37thInternational Convention on Information and Communication Technology.Opatija: Electronics and Microelectronics (MIPRO). p. 653–656 (2014).

[15] Kuhn S, Frankenhauser S, Tolks D (2018). Digital learning and teaching in medical education: already there or still at the beginning?. Bundesgesundheitsblatt Gesundheitsforschung Gesundheitsschutz.

[16] Lai AY, Lee L, Wang MP, Feng Y, Lai TT, Ho LM, Lam VS, Ip MS, Lam TH (2020). Mental health impacts of the COVID-19 pandemic on International University students, related stressors, and coping strategies. Front Psychiatry.

[17] Wang X, Hegde S, Son C, Keller B, Smith A, Sasangohar F (2020). Investigating mental health of US College students during the COVID-19 pandemic: cross-sectional survey study. J Med Internet Res.

[18] Seymour-Walsh AE, Bell A, Weber A, Smith T (2020). Adapting to a new reality: COVID-19 coronavirus and online education in the health professions. Rural Remote Health.

[19] Briano R, Midoro V, Trentin G (1997). Computer mediated communication and online teacher training in environmental education. J Inform Tech Teach Educ.

[20] Trentin G (1999). The quality-interactivity relationship in distance education. Educ Tech.

[21] De Vries L, Naidu S, Jegede O, Collis B (1995). On-line professional staff development: an evaluation study. Distance Education.

[22] Akaslan D, Law EL-C (2011). Measuring Student E-learning Readiness: A Case about the Subject of Electricity in Higher Education Institutions in Turkey. Proceedings of the 10th International Conference on Advances in Web Based Learning (ICWL) Hong Kong, China, LNCS.

[23] Unal S, Alır G, Soydal I (2014). Students readiness for E-learning: an assessment on Hacettepe University Department of information management. Comm Computer Inform Scie.

[24] Balula A, Moreira A. Evaluation of Online Higher Education Learning, Interaction and Technology. Springer (2014).

[25] Blackmon SJ, Major C (2012). Student experiences in online courses: a qualitative research synthesis. Q Rev Dist Educ.

[26] El-Naga NA, Abdulla D (2015). A roadmap to transform learning from face-to-face to online. J Educ Train.

[27] Cramér H (1946). Mathematical methods of statistics.

[28] Goodman LA, Kruskal WH (1954). Measures of association for cross classifications. J Am Stat Assoc.

[29] Kendall MG (1945). The treatment of ties in rank problems. Biometrika..

[30] Pearson K (1900). On the criterion that a given system of deviations from the probable in the case of a correlated system of variables is such that it can be reasonably supposed to have arisen from random sampling. Philos Mag.

[31] Shachar, M. & Neumann, Y. Differences between traditional and distance education academic performances: a meta-analytic approach. The International Review of Research in Open and Distributed Learning Athabasca University Press 4, (2003).

[32] Smith CE, Fontana-Chow K, Boateng BA, Azzie G, Pietrolungo L, Cheng-Tsallis A, Golding F, Tallett S (2009). Tele-education: linking educators with learners via distance technology. Pediatr Ann.

[33] O’doherty D, Dromey M, Lougheed J, Hannigan A, Last J, McGrath D (2018). Barriers and solutions to online learning in medical education–an integrative review. BMC Med Educ.

[34] Gewin V (2020). Five tips for moving teaching online as COVID-19 takes hold. Nature..

[35] Schneider SL, Council ML (2020). Distance learning in the era of COVID-19. Arch Dermatol Res.

[36] Sahi PK, Mishra D, Singh T (2020). Medical education amid covid-19 pandemic. Indian Pediatr.

[37] Cook DA, Dupras DM (2004). A practical guide to developing effective web-based learning. J Gen Intern Med.

[38] Chick RC, Clifton GT, Peace KM, Propper BW, Hale DF, Alseidi AA, et al. Using technology to maintain the education of residents during the COVID-19 pandemic. J Surg Educ. 2020;77(4):729-732. 10.1016/j.jsurg.2020.03.018 PMC7270491 32253133

[39] Mokadam NA, Verrier ED. Flipping the classroom: how to optimize learning in the didactic setting. Hermsen JL, Thorac Surg Clin. 29, 279–284 (2019). 10.1016/j.thorsurg.2019.04.002 31235296

[40] Bediang G, Stoll B, Geissbuhler A, Klohn A, Stuckelberger A, Nko'o S (2013). Computer literacy and e-learning perception in Cameroon: the case of Yaounde. Faculty of Medicine and Biomedical Sciences. BMC Med Edu.

[41] Gillett B, Peckler B, Sinert R, Onkst C, Nabors S, Issley S, Maguire C, Galwankarm S, Arquilla B (2008). Simulation in a disaster drill: comparison of high- fidelity simulators versus trained actors. Acad Emerg Med.

[42] Gagnon K, Young B, Bachman T, Longbottom T, Severin R, Walker MJ (2020). Doctor of physical therapy education in a hybrid learning environment: reimagining the possibilities and navigating a "new Normal". Phys Ther.

