# Correction to: COVID-19 and dental distance-based education: students’ perceptions in an Italian University

**DOI:** 10.1186/s12909-021-02971-7

**Published:** 2021-10-12

**Authors:** Paola Di Giacomo, Carlo Di Paolo

**Affiliations:** Department of Oral and Maxillo-facial Sciences, Sapienza University of Rome, Via Caserta 6, 00161 Rome, RM Italy

**Correction to: BMC Med Educ 21, 414 (2021)**

**https://doi.org/10.1186/s12909-021-02840-3**

Following publication of the original article [1], the authors identified an error in the author name of Paola Di Giacomo.

The incorrect author name is: Paolo Di Giacomo

The correct author name is: Paola Di Giacomo

The author group has been updated above and the original article [1] has been corrected.
