# Supplementary material for: COVID-19 and dental distance-based education: students’ perceptions in an Italian University
Source: BMC Med Educ. 2021 Aug 2;21:414. doi: 10.1186/s12909-021-02840-3 (PMC8327040; doi:10.1186/s12909-021-02840-3)
Supplement: Supplementary file 1 — Additional file 1. Covid-19 and distance learning questionnaire. [file 12909_2021_2840_MOESM1_ESM.docx]

Covid-19 and distance learning questionnaire.

| 1. **Indicate the faculty you are attending (Choose one of the alternatives).**  - Dentistry - Oral Hygiene |
| --- |
| 1. **Indicate the year of your course.** |
| 1. **Sex.** |
| 1. **Age.** |
| 1. **Was the learning environment adequate ( for example if it is noisy, make yourself comfortable, etc)? (Choose one of the alternatives).**  - Yes - No - Quite enough |
| 1. **Was your technology (computer, internet connection) adequate to attend distance lessons? (Choose one of the alternatives).**   - yes  - No  - quite enough |
| 1. **Was the online platform adequate? (Choose one of the alternatives).**  - Yes - No - Quite enough |
| 1. **The quality of lessons has… (Choose one of the alternatives).**  - Remained unchanged - Improved - Worsened |
| 1. **How often did your professors perform traditional lessons with slides?**   (Choose a score from 0 to 5, where 0= never and 5= always) |
| 1. **How often did your professors perform lessons in which you had previously received educational support materials (flipped classroom)?**   (Choose a score from 0 to 5, where 0= never and 5= always) |
| 1. **How often did your professors perform lessons in which you could participate in problem solving of clinical cases?**   (Choose a score from 0 to 5, where 0= never and 5= always) |
| 1. **Did your professors provide you additional materials to deepen your lessons (such as slides, scientific manuscripts, clinical cases)? (Choose one of the alternatives).**  - Yes, more than 50% of professors - Yes, less than 50% of professors - No |
| 1. **During distance lessons, professors managed to promote my attention… (Choose one of the alternatives).**  - Never - Sometimes - Often - Always |
| 1. **How many hours on average per week did you attend distance lessons?** |
| 1. **Follow online lessons was…. (Choose one of the alternatives).**  - More comfortable - More relaxing - No differences with in-class lessons |
| 1. **At the end of day, compared to in-class lessons, physical fatigue has…. (Choose one of the alternatives).**  - Remained unchanged - Improved - Worsened |
| 1. **During online lessons… (Choose one of the alternatives).**  - I could stay focused - I occasionally got distracted - I distracted myself more than during traditional lessons |
| 1. **Compared to in-class lessons the possibility to interact with the professors has…. (Choose one of the alternatives).**  - Remained unchanged - Improved - Worsened |
| 1. **The quality and quantity of knowledge you could acquired, compared to in-class lessons has …. (Choose one of the alternatives).**  - Remained unchanged - Improved - Worsened |
| 1. **Are you afraid that your level of knowledge could decrease by continuing distance lessons ? (Choose one of the alternatives).**  - Yes - No - I don’t know |
| 1. **What is your overall level of satisfaction about distance education?**   (Choose a score from 0 to 10, where 0= I am not satisfied and 10= I am very satisfied) |
| 1. **What would you change to improve your distance lessons?** |
| 1. **What was the best aspect of distance education?** |
| 1. **What was the worst aspect of distance education**? |
